# 

**Published:** 1787

**Authors:** 


### CATALOGUE OF BOOKS.

1. AN Eſſay on difficult Labours. Part I. By *Thomas Denman*, M. D. Licentiate in Midwifery of the College of Phyſicians. 8vo. *Johnſon*, London, 1787.

2. Practical Obſervations on the Natural Hiſtory and Cure of the Venereal Diſeaſe; in three volumes. By *John Howard*, Surgeon. 8vo. *Baldwin*, London, 1787.

3. A Treatiſe on the Venom of the Viper; on the American Poiſons; and on the Cherry Laurel, and ſome other Vegetable Poiſons: to which are annexed, Obſervations on the primitive ſtructure of the Animal Body; different Experiments on the reproduction of the Nerves; and a deſcription of a new Canal of the Eye. Tranſlated from the original French of *Felix Fontana*, Naturaliſt to his R. H. the Grand Duke of Tuſcany, and Director of his Cabinet of Natural Hiſtory. By *Joſeph Skinner*, Navy Surgeon, and Member of the Corporation of Surgeons of London. 8vo. *Murray*, London, 1787. 2 vols.

4. Phyſiological Conjectures concerning certain functions of the Human Economy in the

Fœtus

Fœtus and the Adult. By *James Rymer*, 8vo. *Evans*, London, 1787.

5. Memoirs of the Medical Society of London, inftituted in the year 1773. Vol. 1. 8vo. *Dilly*, London, 1787.

6. An Extraordinary Cafe of lacerated Vagiga, at the full period of Geftation; with Obfervations, tending to fhow that many Cafes related as Ruptures of the Uterus, have been lacerations of the Vagina, By *William Goldfon*, Member of the Corporation of Surgeons in London. 8vo. *Murray*, London, 1787.

7. An Eftimate of the Temperature of different Latitudes. By *Richard Kirwan*, Efq. F.R.S. and Member of the Academies of Stockholm, Upfal, Dijon, Dublin, Philadelphia, &c. 8vo. *Elmfly*, London, 1787.

8. An Effay on Phlogifton, and the Conftitution of Acids. By *Richard Kirwan*, Efq. F.R.S. &c. 8vo. *Elmfly*, London, 1787.

9. A Lecture containing plain Defcriptions of the fituation of the large Blood Veffels of the extremities; the Inftrument called Tourniquet; and the methods of making effectual preffure on the Arteries, in cafes of dangerous effufions of Blood from Wounds, &c. Delivered to the Scholars of the Maritime School at at Chelſea. Firſt printed for their uſe, and now publiſhed for general benefit, by *William Blizard*, F. A. S. Surgeon to the London Hoſpital, and the Hon. Artillery Company, and Lecturer in Anatomy and Surgery. 8vo. *Dilly*, London, 1786.

10. Experiments and Obſervations on the danger of Copper, and Bell Metal, in Pharmaceutical and Chemical preparations. By *William Blizard*, F. A. S. 8vo. *Dilly*, London, 1786.

11. The Hiſtory of ſome remarkable Cures, in Worm Caſes, by a mild and efficacious Medicine, which ſtrengthens the Conſtitution, without any preparation of Mercury or Tin; and proves that all Complaints occaſioned by Worms, may be cured without uſing of violent Medicines, which always injure the Conſtitution of the Patient. Alſo, a certain Method of curing that obſtinate Conſtipation of the Bowels, commonly called the Dry Gripes, in a ſhort time, frequently leſs than twenty-four hours; which Painters, Plumbers, and all workers of White Lead, are grievouſly afflicted with. By *J. Harriſon*, Member of the Corporation of Surgeons, in London. *Faulder*. 8vo. London, 1786.

12. A Treatiſe on the Cauſes and Effects of Schirrous Tumours and Cancers. To which are annexed, an addrefs to the Public, and the Authors Apology, for introducing to the world the late Mr. Guy's Arcanum, refpecting the above diforders; together with teftimonials of his fuccefs; evincing from reafon and experience, that application to be the only one extant, capable of effectually removing all Schirrous Tumours, without the knife. By *Henry Saffory*, Member of the Corporation of Surgeons, London, and late Surgeon of Light Dragoons, in America. 8vo. *Lane*, London, 1786.

13. An Oration delivered before the American Philofophical Society, held in Philadelphia, on the 27th of February, 1786; containing an Inquiry into the influence of Phyfical Caufes upon the Moral Faculty. By *Benjamin Rufh*, M. D. and Profeffor of Chemiftry in the Univerfity of Pennfylvania, 4to. Philadelphia, 1786.

14. A Synopfis of a Courfe of Lectures on the Theory and Practice of Medicine, in four Parts. Part I. By *B. Waterhoufe*, M. D. Profeffor of the Theory and Practice of Phyfic in the Univerfity of Cambridge, New England; and of Natural Hiftory in the College of Rhode Ifland. 8vo. Bofton, 1786.

15. Differtatio

15. Diſſertatio Inauguralis, ſiſtens experimenta chemica, et inſtrumenta chirurgica emendata. Auctore *Carol. Henric. Bernh. Weigel*, 4to. Gryphiæ, 1785.

16. Programma, quo *Chriſt. Ehrenfr. Weigel*, Diſſertationem *C. H. B. Weigel*, publice defendendam indicit, præmittens Hiſtoriæ Baryllio- rum rudimenta. 4to. Gryphiæ, 1785.

17. Sermo Academicus de Civis Medici in Republica conditione atque officiis ex lege præcipue erutis, quem profeſſionis Medicæ adeundæ cauſſa die 24. Novembr. 1785. *I. P. Frank*, M. D. et in Reg. Ticin. Academia Med. Pract. Prof. 8vo. Pavia, 1785.

18. *I. P. Frank* Oratio Academica, de veſica urinali ex vicinia Morboſa ægrotante, Die 29, Aprilis, 1786, recitata. 8vo. Pavia, 1786.

19. *Franciſci Tavares*, M. D. in Conimbric. Univers. P. P. O. &c. de Pharmacologia Libellus Academicis Prælectionibus accomodatus. 8vo. Coimbra, 1786.

20. Synopſis Noſologiæ Methodicæ, continens Genera Morborum. Auctore *Gulielmo Cullen*, M. D. editio quarta, emendata et plurimum aucta. Recudi curavit et præfatus eſt *J. P. Frank*, M. D. &c. 8vo. Pavia, 1787.

21. Diſſertatio Inauguralis Medica de Vermibus medicatis, Auctore *Joanne Juſto Guilielmo Forcke*, Springa-Hannoverano. 4to. Goettingæ, 1786.

22. Diſſertatio Medica de Medicina Africanorum, quam præſide *Carol. P. Thunberg*, M. D. Prof. Med. et Botan. Reg. et Ord. &c. pro gradu Doctoris publico examini ſubjicit *Petrus Ulr. Berg*. Uplandus, 1785.

23. Diſputatio Botanica de Erica, quam præſide *Carol. P. Thunberg*, Med. Doct. Prof. Med. et Botan. &c. publico examini ſubjicit *Jacobus Bernhardus Struve*, Veſtro-Gothus. 4to. Upſaliæ, 1785, c. tab. æn. 6.

24. Diſſertatio Botanico-Medica de Aloe, quam præſide *C. P. Thunberg*, M. D. Prof. Med. et Botan &c. publico examini ſubjicit *Andreas Heſſelius*, V. Gothus, 4to. Upſaliæ, 1785.

25. Exercitationes Phyſico-Medicæ de admiranda Naturæ ſimplicitate, et de utili quidem, ſed admodum limitanda, Medicina populari, auctore *Leon. Ludov. Finke*, M. D. et Prof. in Acad. Lingenſi. 8vo. 1785.

26. Hiſtoria Salicum iconibus illuſtrata a *Georgio Franciſco Hoffman*. Faſcic. I. II. III. Folio. Lipſiæ, 1785-6.

27. De

27. De Violæ Caninæ in Medicina uſu, auctore *Joanne Henrico Andrea Niemeyer*, Nordheimenſi. 4to. Goettingæ, 1785.

28. Methodus Formulas Medicas conſcribendi; in uſum prælectionum Academicarum edidit *Jo. Frid. Pichler*, M. D. et Coll. Med. Argentorati Socius. 4to. Argentor. 1785.

29. *Geo. Rud. Boehmeri*, Bibliotheca Scriptorum Hiſtoriæ Naturalis, Oeconomiæ, aliarumque artium ac Scientiarum ad illam pertinentium realis ſyſtematica. Tom. I. 8vo. Lipſiæ. 1785.

30. Remede nouveau, contre les Maladies veneriennes, tiré du regne Animal; ou Eſſai ſur la vertu anti-venerienne des Alkalis volatils; dans lequel on expoſe la methode d'adminiſtrer ces ſels; avec des reflexions, des obſervations, et des remarques critiques tendantes a perfectionner les autres methodes. Par M. *Bern. Peyrilhe*, D. M. Profeſſeur Royal de Chimie et de Botanique au College de Chirurgie de Paris, &c. Seconde edition, revue et conſiderablement augmentée. 8vo. Montpellier, 1786.

31. Eſſai d'un Syſteme des Tranſitions de la Nature dans le regne Mineral. Par M. le Comte

Comte G. *de Razoumowsky*, Membre de la Société de Lausanne, &c. 8vo. Lausanne, 1785.

32. Les Regles et les Preceptes de la Santé, de Plutarque, traduits du Grec par *Jacques Amyot*, Grand Aumonier de France, avec des Notes et des Observations de M. l'Abbé *Brotier*. 12mo. Paris, 1785.

33. Project d'Instruction sur une Maladie convulsive, frequente dans les Colonies de l'Amerique, connue sous le nom de Tetanos. Demandé par le Ministre de la Marine à la Société Royale de Medecine. 8vo. Paris, 1786.

34. L'Art de Prolonger la Vie, et de Conserver la Santé ; ou Traité d'Hygiene. Par M. *Pressavin*, Gradué de l'Université de Paris, Membre du College Royal de Chirurgie de Lyon, et ancien Demonstrateur en Matiere Medico-Chirurgicale. 8vo. Lyon, 1784.

35. Memoire qui a remporté le Prix au jugement de l'Academie de Dijon en 1782, sur la question proposée en ces termes : determiner avec plus de precision qu'on ne l'a fait jusqu'a present, le caractere des fievres intermittentes, et indiquer par des signes non equivoques, les circonstances dans lesquelles les febrifuges peuvent etre employés avec avantage, et sans danger, pour les malades. Par M.

*Voullonne*

*Voullonne*, Docteur en Medecine de la Faculté de Montpellier, Agrégé et premier Professeur dans la Faculté d'Avignon. 8vo. Avignon, 1786.

36. Nouvelles instructives Bibliographiques, historiques, et critiques, de Medecine, Chirurgie et Pharmacie; ou Recueil raisonné de tout ce qu'il importe d'apprendre chaque année pour etre au courant des connoissances et à l'abri des erreurs relatives a l'art de guerir. Années 1785, 86, et 87. Tom. III. 12mo. Paris, 1785-6-7.

37. Examen d'un Ouvrage intitulé Nouvelles instructives, bibliographiques, historiques et critiques de Medecine, Chirurgie, Pharmacie, &c. Par M. *Pierre Sue*, ancien Prevot du College de Chirurgie, &c. 8vo. Paris, 1786.

38. Ueber die Regeneration der Nerven. Ein Brief an Herrn Peter Camper. *i. e.* On the Regeneration of Nerves. A letter to M. Peter Camper. By *Frederick Michaelis*, M. D. &c. 8vo. Cassel, 1785.

39. Ueber anwendung der Elektricitaet bey Kranken, nebst der beschreibung der neuen Maschine von Nairne, zur positiven und negativen Elektricitaet; auch eines neuen Elektrischen Bettes. *i. e.* On the Application of Electricity to the Sick; with a Description of Nairne's

Nairne's new Machine for poſitive and negative Electricity; and likewiſe of a new Electrical Bed. By *J. L. Boeckmann*, Profeſſeur of Natural Philoſophy at Carlſruhe. 8vo. Durlach, 1786; with a Plate.

40. Theoretiſche und practiſche Bemerkungen ueber des Muſkelvermoegen der haargefaeſſchen; nebſt einigen anwendungen deſſelben zur erklaerung einiger erſcheinungen in dem geſunden und kranken thieriſchen Koerper. *i. e.* Theoretical and practical Obſervations on the Muſcular power of the Capillary Veſſels; with ſome applications of it to the explanation of certain appearances in the healthy and diſeaſed Animal body. By *H. Van der Boſch.* 8vo. Munſter. 1786.

41. Ueber den gegenwaertigen mangel guter Wundaerzte und Geburtshelfer in dem groeſſten Theile Deutſchlands, nebſt einigen Vorſchlaegen, diesem mangel abzuhelfen. *i. e.* on the preſent ſcarcity of good Surgeons and Accoucheurs in the greater part of Germany, with ſome propoſals to remedy this defect. By G. *F. B. Raven*, Surgeon and Accoucheur at Zell. 8vo. Goettingen. 1786.

42. Inſtruzioni Mediche per le genti di Campagna, 8vo. Baſſano. 1785.

43. Inſtruzione veterinaria pe maniſcalchi, e coloni ſulla preſente epidemia contagioſa de' buoi limitrofa all 'agro Riminese ; compoſta, e corredata di note dal Conte *Franceſco Bonſi.* 8vo. Rimino. 1786.

44. Odontologia, oſſia trattato ſopra i denti : opera di *A. Campani*, publico Dentiſta Florentino, &c. 8vo. Firenze, 1786.

45. Diſſertazione ſopra l'origine delle malattie, e ſopra il loro ſpecifico rimedio, aggiuntovi il metodo di adoperarlo. 8vo. Roma, 1787.

46. Eſſai ſur le Lait conſideré medicinalement ſous ſes differens aſpects, ou Hiſtoire de ce qui a rapport à ce fluide chez les femmes, les enfans et les adultes, ſoit qu'on le regarde comme cauſe de maladie, comme aliment, ou comme medicament. Par M. *Petit-Radel*, Docteur Regent de la Faculté de Medecine de Paris, et ancien Chirurgien Major du Roi dans les Indes Orientales. 8vo. Paris, 1786.

47. Recherches ſur la cauſe des Affections hypocondriaques, appelées communement vapeurs ; ou Lettres d'un Medecin ſur ces affections. On y a joint un Journal de l'Etat du corps en raiſon de la perfection de la tranſpiration et de la temperature de l'air. Par M.

*Claude Revillon*, D. M. de l'Academie des Sciences de Dijon, Correſpondant de la Société Royale de Medecine de Paris, à Macon. Nouvelle edition. 8vo. Paris, 1786.

48. Eſſais ſur l'Hiſtoire Medico-topographique de Paris, ou Lettres a M. d'Aumont, Profeſſeur en Medecine a Valence, ſur le Climat de Paris, ſur l'Etat de la Medecine, ſur le Caractere et le Traitement des Maladies, et particulierement ſur la petite verole et l'Inoculation. Par M. *Menuret de Chambaud*, D. M. de l'Univerſité de Montpellier, Medecin Conſultant de Madame la Comteſſe d'Artois, &c. 12mo. Paris, 1786.

49. Traité des Maladies des yeux et des Oreilles, conſiderées ſous le rapport des quatres parties ou quatre ages de la vie de l'homme, avec les remedes curatifs, et les moyens propres a les preſerver d'accidens; avec Planches gravées en taille douce. Par M. l'Abbé *Deſmonceaux*. 2 Tomes. 8vo. Paris, 1786.

50 Traité de l'Hydrocele, cure radicale de cette Maladie, et traitement de pluſieurs autres. Par M. *Imbert Delonnes*, premier Chirurgien de Mgr. le Duc d'Orleans, &c. 8vo. Paris, 1786.

51. Nou-

51. Nouvelles Obſervations pratiques ſur les maladies de l'oeil et leur traitement; ouvrage fondé ſur une nouvelle theorie, dans lequel l'auteur explique et concilie pluſieurs methodes d'operer la cataracte, et propoſe differens inſtrumens nouveaux pour cette operation, ainſi que pour les diverſes maladies qui affectent l'oeil. Par M. *Gleize*, D. M. Medecin Oculiſte de Monſeigneur le Comte d'Artois. 8vo. Paris, 1786.

52. Recherches phyſiologiques et philoſophiques ſur la ſenſibilité ou la vie animale. Par M. *de Seze*, D. M. de l'Univerſité de Montpellier, agrégé à la Faculté de Bordeaux. 8vo. Paris, 1786.

53. Eſſai ſur le Fluide Electrique, conſideré comme agent univerſel. Par feu M. le Comte *de Treſſan*, Lieutenant General des Armées du Roi, Membre des Academies Royales des Sciences de Paris, Londres, &c. 8vo. 2 Tomes. Paris, 1786.

54. Maniere d'allaiter les enfans à la main au defaut de Nourrice; traduit de l'Italien de M. *Baldini*. 8vo. Paris, 1786.

55. Bemerkungen ueber die enſtehung und behandlung verſchiedener arten von fiebern. *i. e.* Obſervations on the Origin and Treatment of different Species of Fevers. By *Chr. Fred. Richter*, M. D. 8vo. Halle, 1785.

56. Praktische Abhandlung einiger das Nervensystem betreffenden Krankheiten. *i. e.* A practical Essay on some Diseases of the nervous System. By *J. G. Khun*, M. D. 8vo. Bresslaw, 1786.

57. Abhandlungen der Boehmischen Gesellschaft der Wissenschaften auf das Jahr 1785. *i. e.* Transactions of the Bohemian Society of Sciences for the Year 1785. 4to. Prague, 1785.

58. Kleine physikalisch-chemische Abhandlungen. *i. e.* Physico-chemical Essays. By *John Fred. Westrumb*. 8vo. Leipsic, 1785.

59. Rapsodien der philosophischen Pharmakologie; nebst einer Anleitung zur theoretisch-praktischen Chemie und einer Tabelle ueber die experimental Pharmacie. *i. e.* Rhapsodies of philosophical Pharmacology; with a Plan of theoretico-practical Chemistry, and a Table relative to experimental Pharmacy. By *J. J. Bindheim*. 8vo. Berlin, 1785.

60. Abhandlung von einer neuen Methode die hartnaeckigsten Krankheiten, die ihren sitz im Unterleibe haben, besonders die Hypochondrie, sicher und gründlich zu heilen. *i. e.*

Essay

Eſſay on a new Method, by Means of which the moſt obſtinate Diſeaſes that have their Seat in the lower Belly, particularly the hypochondriacal Affection, may be ſafely and radically cured. By *John Kæmpf*, M. D. Body Phyſician to the Prince of Heſſe-Hanau, &c. 8vo. Deſſau, 1784.

61. D. *Gualth. Van Doeveren* Primæ Lineæ de cognoſcendis mulierum morbis in uſus Academicos; recudi curavit D. *Joh. Chriſt. Traugott Schlegel*, Medicus apud Longoſaliſſenſes. 8vo. Lipſiæ, 1786.

62. *A. Cornelii Celſi* Medicinæ libri octo ex recenſione *Leonardi Targæ*; accedunt notæ variorum, item quæ nunc primum prodeunt, *J. L. Bianconii* Diſſertatio de Celſi ætate, et *Ge. Matthiæ* Lexicon Celſianum. 4to. Lugd. Bat. 1785.

63. *Henrici Auguſti Wriſberg*, Phil. et Med. Doct. Conſil. Aulici Regii et Electoralis, Med. et Anat. in Univerſ. Georgia Auguſta Profeſſ. public. et ordin. &c. Sylloge Commentationum Anatomicarum, 1. De membranarum ac involucrorum continuationibus; 2. De nervis arterias venaſque comitantibus; et 3. De nervis pharyngis. 4to. Goettingæ, 1786.

64 *Hen-*

64. *Henrici Friderici Delii*, Conf. intim. Aulæ Brandenb. Medic. Profeff. primar. Adverfaria argumenti phyfico-medici. 4to. Erlangæ, 1785.

65. *Jofephi Jacobi Plenk*, Chirurg. Doct. Chemiæ atque Botanices Prof. publ. ordin. in Acad. Chirurg. Milit. &c. Toxicologia, five Doctrina de Venenis et Antidotis. 8vo. Viennæ, 1785.

66. Ad audiendam orationem follemnem, qua ordinariam Academiæ Profeffionem 1785, aufpicaturus eft, humaniffime invitat *Chr. G. Efchenbach*, præmittuntur de quibufdam auri calcibus et falibus mercurialibus obfervationes. 4to. Lipfiæ, 1785.

67. *Johannes Gottlieb Walter* de morbis Peritonæi et Apoplexia. Cum Tabulis Æneis. 4to. Berolini, 1785.

68. Carmen de Medico, ignorata morbi cauffa, male curante. 8vo. Tubingæ, 1784.

69. Difcorfo Academico dei Vantaggi della Educazione filofofica nello Studio della Chimica. *i. e.* An academic Difcourfe on the Advantages of a philofophical Education in the Study of Chemiftry. By *Petr. Mofcati*, Regius Profeffor of Surgery and Chemiftry. 8vo. Milan, 1784.

70. Pro-

70. Prodrome d'un Ouvrage ſur le Syſteme des Vaiſſeaux lymphatiques contenant vingt quatre planches in Folio, par *Paul Maſcagni*, Profeſſeur d'Anatomie dans l'Univerſité de Sienne. 4to. Sienne, 1784.

71. Manuel des Goutteux et des Rhumatiſtes; ou l'art de ſe traiter ſoi meme de la Goutte, du Rhumatiſme, et de leur complication, avec la maniere de s'en preſerver, de s'en guerir, et d'en eviter la recidive. Par M. *Gachet*, Maitre en Chirurgie. 8vo. Paris, 1786.

72. Manuel pour le ſervice des Malades, ou Precis des Connoiſſances neceſſaires aux perſonnes chargées du ſoin des malades, femmes en couches, enfans nouveau-nés, &c. Par M. *Carrere*, Conſeiller Medecin ordinaire du Roi, Profeſſeur Royal emerite en Medecine, Cenſeur Royal, ancien Inſpecteur General des Eaux Minerales du Rouſſillon et du Comté de Foix, ci devant Directeur du Cabinet d'Hiſtoire naturelle de l'Univerſité de Perpignan, de la Société Royale de Medecine, des Academies de Toulouſe, de Montpellier, des Curieux de la Nature, &c. 12mo. Paris, 1786.

73. Traité d'Anatomie et de Phyſiologie, avec des planches Coloriées, repreſentant au naturel les divers organes de l'hommé et des animaux, animaux, dedié au Roi; par M. *Vicq D'Azyr*, Docteur regent et ancien Professeur de la Faculté de Medecine a Paris, de l'Academie Royale des Sciences, Secretaire perpetuel de la Société Royale de Medecine, &ç. Tome I. Folio. Paris, 1786.

74. Recherches sur l'origine et le siege du Scorbut, et des fievres putrides; ouvrage traduit de l'Anglois de M. *Milman*, par M. *Vigaroux de Montagut*, D. M. et Membre de la Société Royale des Sciences de Montpellier. 8vo. Paris, 1786.

75. Cours de Matiere Medicale de M. *Cullen*, D. M. ancien Professeur de Medecine clinique, de Chemie, de Matiere Medicale, &c. dans l'Université d'Edinbourg, mis 'a la portée de la bonne Education; traduit de l'Anglois, pour servir d'Introduction a ses Elemens de Medecine pratique, auquel ou a ajouté des Notes et des Observations. Par M. *Caulet de Veaumorel*, Medecin de la Maison de Monsieur Frere du Roi. Tome I. 8vo. Paris, 1787.

